# Isolation and Characterization of NDM-Positive Escherichia coli from Municipal Wastewater in Jeddah, Saudi Arabia

**DOI:** 10.1128/AAC.00236-16

**Published:** 2016-08-22

**Authors:** David Mantilla-Calderon, Muhammad Raihan Jumat, Tiannyu Wang, Pugalenthi Ganesan, Nada Al-Jassim, Pei-Ying Hong

**Affiliations:** a King Abdullah University of Science and Technology (KAUST), Water Desalination and Reuse Center (WDRC), Biological and Environmental Sciences & Engineering Division (BESE), Thuwal, Saudi Arabia; b King Abdullah University of Science and Technology (KAUST), Bioscience Core Labs, Thuwal, Saudi Arabia

## Abstract

The emergence of resistance to last-resort antibiotics is a public health concern of global scale. Besides direct person-to-person propagation, environmental pathways might contribute to the dissemination of antibiotic-resistant bacteria and antibiotic resistance genes (ARGs). Here, we describe the incidence of *bla*_NDM-1_, a gene conferring resistance to carbapenems, in the wastewater of the city of Jeddah, Saudi Arabia, over a 1-year period. *bla*_NDM-1_ was detected at concentrations ranging from 10^4^ to 10^5^ copies/m^3^ of untreated wastewater during the entire monitoring period. These results indicate the ubiquity and high incidence of *bla*_NDM-1_ in the local wastewater. To track the bacteria carrying *bla*_NDM-1_, we isolated Escherichia coli PI7, a strain of sequence type 101 (ST101), from wastewater around the Hajj event in October 2013. Genome sequencing of this strain revealed an extensive repertoire of ARGs as well as virulence and invasive traits. These traits were further confirmed by antibiotic resistance profiling and *in vitro* cell internalization in HeLa cell cultures. Given that this strain remains viable even after a certain duration in the sewerage, and that Jeddah lacks a robust sanitary infrastructure to fully capture all generated sewage, the presence of this bacterium in the untreated wastewater represents a potential hazard to the local public health. To the best of our knowledge, this is the first report of a *bla*_NDM-1_-positive E. coli strain isolated from a nonnosocomial environment in Saudi Arabia and may set a priority concern for the need to establish improved surveillance for carbapenem-resistant E. coli in the country and nearby regions.

## INTRODUCTION

The overuse of antibiotics in clinical settings and as prophylactics in livestock production has led to the ongoing explosion of multidrug-resistant pathogens, which in conjunction with the low discovery rates of new antibiotics threatens the advent of a postantibiotic era (1). Recent epidemics of methicillin-resistant Staphylococcus aureus (MRSA) infections (2), multidrug-resistant respiratory pathogens (3, 4), and extended-spectrum β-lactamase-producing Escherichia coli and Klebsiella pneumoniae (5) are a few examples of the worldwide proliferation of antimicrobial resistance. This global crisis over antibiotic resistance is further aggravated by the relatively recent discovery of the New Delhi metallo-β-lactamase enzyme (NDM), which confers resistance to imipenem and meropenem, two important carbapenems used as antibiotics of last resort against Gram-negative bacterial infections (6). Since it was first reported on the Indian subcontinent, NDM has become a global public health concern, as NDM-positive bacteria rapidly spread to different regions in the world. So far, carbapenem resistance conferred by the presence of the *bla*_NDM-1_ gene has been widely reported across the globe, with most of these reports corresponding to clinical isolates (7, 8) and only a few studies focusing on the isolation of NDM-positive environmental bacteria (9, 10).

Besides direct person-to-person dissemination of antibiotic-resistant bacteria (ARB) and antibiotic resistance genes (ARGs) in hospital and community settings, these emerging biological contaminants are also mobilized via environmental pathways. The characterization of these environmental isolates may provide information regarding the underlying mechanism favoring the successful environmental persistence of multidrug-resistant pathogens. Special attention has been given to wastewater due to the high abundances of ARB and ARGs deriving from human and animal feces and the presence of subtherapeutic antibiotic and heavy metal concentrations that favor the maintenance and proliferation of antimicrobial resistance in these aquatic ecosystems (11 – 13). Other human activities, such as international tourism and pilgrimage, further contribute to the spread of these emerging biological pollutants, as visitors might carry multiresistant infectious agents that can ultimately be disseminated via the local sewage system (14). This is of special relevance for Saudi Arabia, with Mecca and its neighboring cities serving as locations for Hajj and Umrah pilgrimages, both of which are carried out by approximately 2 million pilgrims of over 80 different nationalities per year (15).

Jeddah City, Saudi Arabia, constitutes the main entry point for pilgrims intending to perform Hajj or Umrah. As of now, only 50% of Jeddah is connected to a centralized wastewater treatment plant (WWTP), while the remaining 50% relies on septic tanks for waste management and disposal (16). Septic tanks are designed to discharge partially treated wastewater into the surrounding area after a certain period. Due to the overuse of septic tanks in many areas of Saudi Arabia, it has been estimated that partially treated wastewater discharged from these septic tanks has caused the groundwater level to rise by up to 0.41 m between 1996 and 2000, in turn suggesting the extent of contamination to the nearby surroundings arising from partially treated wastewater. The potential inflow of emerging pathogens facilitated by the pilgrimage activities may impose a risk to public health, as the country lacks a robust sanitary infrastructure that achieves effective containment of pathogens. The incidence of ARGs conferring resistance to last-resort antibiotics in Saudi wastewater is of special interest, as pathogens resistant to these antibiotics may result in increased mortality or morbidity upon host infection and are likely to be associated with nosocomial infections (17).

Here, we examined the prevalence of the *bla*_NDM-1_ gene over a period of 1 year in the influent wastewater of a WWTP located in Jeddah. Additionally, we described the isolation and characterization of the multidrug-resistant and NDM-1-positive Escherichia coli strain PI7, a potential human pathogen recovered from untreated wastewater in Jeddah. This isolate carries a copy of a plasmid that shares high similarity with the pKOX-NDM1 plasmid, which was first identified in Taiwan in a multidrug-resistant Klebsiella oxytoca clinical isolate (18). The environmental occurrence of this clinically relevant strain hints at the importance of environmental pathways for the mobilization of antibiotic-resistant bacteria.

## MATERIALS AND METHODS

### Wastewater sample collection.

Wastewater was sampled from a WWTP located in Jeddah, Saudi Arabia (19). The amount of wastewater that flowed through this WWTP was 17,700 m^3^/day, which corresponds to roughly 3% of the total wastewater treatment capacity of the city. Untreated wastewater (i.e., influent) was collected after the primary clarifier and before the activated sludge treatment unit on 8 October 2012, 3 December 2012, 21 April 2013, 3 September 2013, and 8 October 2013. Hajj timings correspond to 25 October 2012 and 14 October 2013. Influent samples were collected in 1-liter sterile bottles. Prior to collection, sampling bottles were rinsed twice with their corresponding wastewater samples and immediately transported to the laboratory and stored at 4°C. Samples were processed under aseptic conditions in the subsequent 12 h after collection for cultivation and DNA extraction.

### DNA extraction and *bla*_NDM-1_ detection in wastewater.

DNA extraction was executed as described in a previous study (19). *bla*_NDM-1_ copy numbers were determined by absolute quantification on an Applied Biosystems 7900HT fast real-time PCR system (Thermo Fisher Scientific, Carlsbad, CA, USA) using the TaqMan probe reporter assay. Descriptions of the standard curves, quality control, and protocol related to the real-time PCR procedure are provided in Text S1 and Fig. S1 in the supplemental material.

### Meropenem-resistant bacterial isolation from raw wastewater.

Untreated wastewater collected on 8 October 2013 was chosen for the isolation of NDM-positive bacteria as this date coincided most closely with the influx of Hajj pilgrims. Thirty milliliters of raw municipal wastewater was filtered through a 0.4-μm Whatman Nuclepore track-etched polycarbonate membrane filter (GE Healthcare Life Sciences, United Kingdom). The filter was subsequently placed on a MacConkey agar plate supplemented with meropenem at a final concentration of 8 μg/ml and incubated overnight at 37°C. Pure cultures of the isolates growing in the MacConkey agar plate were established, and their identities were determined by 16S rRNA gene sequencing (20). The presence of extended-spectrum beta-lactamases (ESBL) or carbapenem resistance genes was determined as previously described by Zowawi et al. (21). The activity of NDM was evaluated by the meropenem-EDTA combined disk diffusion method, and the presence of *bla*_NDM-1_ was further confirmed by endpoint PCR (see Text S2 in the supplemental data).

### MLST.

Seven housekeeping genes were used for the E. coli multilocus sequence typing (MLST) analysis (*adk*, *fumC*, *gyrB*, *icd*, *mdh*, *purA*, and *recA*), as previously described (22). PCR products were sequenced in the KAUST Genomics core lab on an ABI 3730XL DNA analyzer (Thermo Fisher Scientific, Carlsbad, CA, USA). Sequence types (STs) were identified using the MLST database of the University of Warwick (http://mlst.warwick.ac.uk/mlst/dbs/Ecoli).

### Genome and plasmid sequencing.

Genomic DNA (gDNA) and plasmid DNA (pDNA) of E. coli PI7 that were submitted for next-generation sequencing were first isolated using a DNeasy blood and tissue kit (Qiagen, Hilden, Germany) and a PureYield plasmid miniprep system (Promega, Madison, WI, USA), respectively. One microgram of gDNA and 1 μg of pDNA were used for genome sequencing on either Illumina MiSeq or Ion PGM Torrent platforms. Raw reads were performed with the initial quality control (QC) checks. Sequences with Phred scores of <20 were removed. Raw reads that passed the QC were then assembled using a CLC genomics workbench (Qiagen, Hilden, Germany) and SOAPdenovo assembly (23), and the resulting contigs were mapped over a reference genome. Detailed descriptions of the bioinformatics QC and assembly steps are provided in Text S3 in the supplemental material.

### Genome and plasmid annotation.

Genome annotation was first carried out with RAST (24). Genomic islands were then predicted with the IslandViewer 3 Web server (25) using as input the annotation generated by RAST. Briefly, IslandViewer uses two algorithms to predict genomic islands; both of them are based on sequence composition methods (SIGI-HMM and IslandPath/DIMOB). SIGI-HMM incorporates synonymous codon usage as the statistical feature. Different species have different preferences for synonymous codon usage; the frequencies at which codons are used do not follow a random distribution but rather constitute a genomic signature. SIGI-HMM derives a codon usage table from the whole-genome sequence of the input organism, and individual gene codon frequencies deviating from the derived whole-genome frequency table are marked as putative foreign genes (26). IslandPath/DIMOB relies (27) on the detection of dinucleotide bias and the presence of at least one mobility gene. Mobility genes are identified based on the genome annotation or by performing comparisons of the predicted genes with mobility gene profiles in Pfam (28). IslandViewer 3 has overall accuracy and recall of 88% and 48%, respectively (25).

Nonclassical secretion signals were identified with Secretome 2.0, in which Gram-positive and Gram-negative secreted proteins are differentiated from cellular proteins by amino acid composition, secondary structure, and disordered regions (29).

### Resistance profiling and MIC determination.

Antimicrobial resistance genes were identified using ResFinder 2.1 (30). Subsequently, antimicrobial resistance was confirmed by establishing the MICs for E. coli PI7 using the microtiter broth dilution method. A detailed description of the MIC determination assays are provided in Text S4 in the supplemental material. A wide range of antibiotics, including gentamicin, ampicillin, kanamycin, ceftazidime, erythromycin, sulfamethoxazole-trimethoprim, chloramphenicol, tetracycline, and meropenem, were tested. The MIC of a particular antibiotic was determined based on the concentration required to achieve ≥70% growth inhibition compared to that of the positive control.

### E. coli PI7 invasiveness test.

Invasiveness assays were performed using (i) E. coli PI7 and DSM 1103 or (ii) a green fluorescent protein (GFP)-tagged E. coli PI7 strain and a GFP-tagged DH5α derivative strain. The procedures to obtain the GFP-tagged strains are described in Text S5 in the supplemental material. Invasion was determined by (i) quantification using plating techniques or (ii) confocal microscopy when infection was performed with the GFP-tagged strain derivatives. Briefly, HeLa cells were seeded in 24-well tissue culture plates at an initial density of 3 × 10^5^ cells/well and incubated overnight at 37°C in 5% CO_2_. The bacterial inoculum was grown on LB broth to an optical density at 600 nm (OD_600_) of 0.8. Bacterial cells were pelleted (5,000 × *g* for 10 min) and resuspended in Dulbecco's modified Eagle's medium (DMEM). Each well was either inoculated independently with 300 μl of the bacterial suspension (*n* = 4 for each strain) or mock infected with sterile DMEM (*n* = 8) and then incubated 37°C in 5% CO_2_ for 2 h (see Text S6 in the supplemental material). Subsequently, HeLa cells infected with the GFP-tagged strains or mock infected (*n* = 4) were washed with 250 μl of 1× phosphate-buffered saline (PBS) and prepared for fixation and staining for confocal microscopy (see Text S7 in the supplemental material). HeLa cells infected with the non-GFP-tagged strains were treated with 200 U of mutanolysin (Sigma-Aldrich, St. Louis, MO, USA). Cells were then incubated at 37°C in 5% CO_2_ for 4 h before being washed twice with sterile 1× PBS. Washed cells were then lysed by 0.2% Triton in 1× PBS for 20 min at 4°C. The lysed contents were harvested and spun down at a low speed of 80 × *g* for 10 min to collect cell debris. The supernatant fraction of the lysate was serially diluted in 1× PBS and spread plated on LB agar plates. LB agar plates used for spread cultivation of E. coli PI7 were supplemented with 8 μg/ml meropenem to ensure no contamination. Bacterial colonies were counted after overnight incubation at 37°C. The entire procedure was repeated twice to obtain three sets of biological replicates.

### Accession number(s).

The SFF files obtained from the E. coli PI7 genome and plasmid sequencing are deposited in the Short Read Archive (SRA) of the European Nucleotide Archive (ENA) under study accession number PRJEB12510.

## RESULTS

### Environmental occurrence of *bla*_NDM-1_ genes in untreated wastewater.

**FIG 1:**
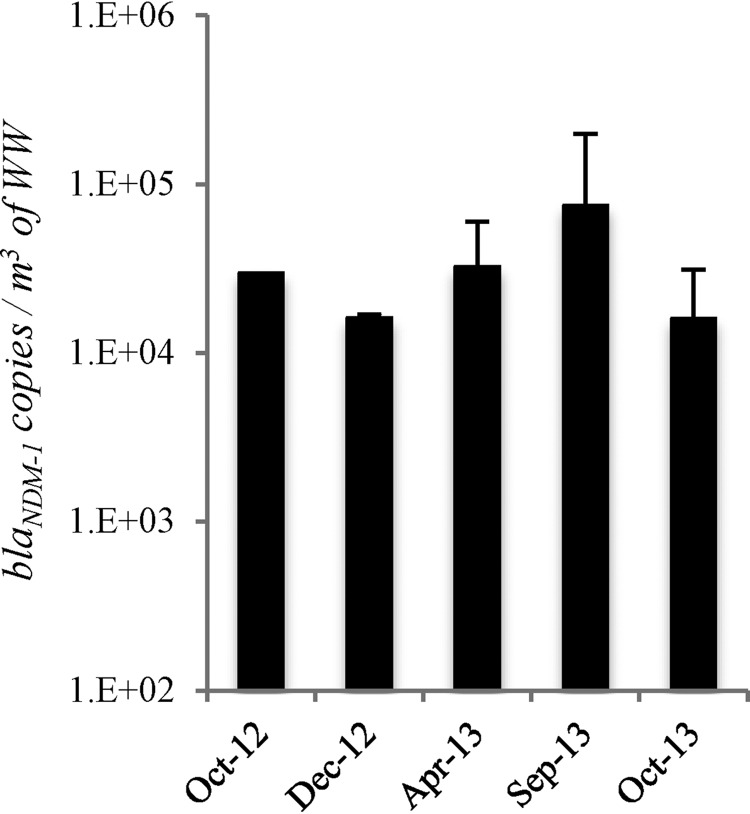
Incidence of *bla*_NDM-1_ in the raw wastewater (WW) of a wastewater treatment plant in Jeddah, Saudi Arabia, over a 1-year period.

Quantitative PCR (qPCR) was performed to determine the abundance of *bla*_NDM-1_ in untreated wastewater received by the local wastewater treatment plant. Over a period of 1 year, *bla*_NDM-1_ was consistently detected in the influent wastewater at an average abundance of 3.4 × 10^4^ ± 2.3 × 10^4^ copies/m^3^ of raw wastewater (Fig. 1). The abundance of *bla*_NDM-1_ fluctuated throughout the year, with the lowest *bla*_NDM-1_ abundance of 1.6 × 10^4^ ± 8.9 × 10^2^ copies/m^3^ observed in December 2012. The abundance of *bla*_NDM-1_ was the highest during September 2013, with an average incidence of 2 × 10^5^ ± 1.8 × 10^4^ copies/m^3^ of wastewater.

### Environmental occurrence of NDM-positive E. coli PI7 from raw wastewater.

In order to further evaluate the risk associated with the high incidence of *bla*_NDM-1_ in the Jeddah wastewater, an isolation effort to recover meropenem-resistant bacteria was carried out. A total of 24 colonies were recovered from 30 ml of influent wastewater in MacConkey agar plates supplemented with meropenem. The identities of these isolates are shown in Table S1 in the supplemental material. In this study, ARB in the wastewater that are opportunistic pathogens (e.g., Aeromonas, Pseudomonas, and Escherichia spp.) and that might represent a direct threat to the public health when released into the environment were further characterized for ESBL or carbapenem resistance genes. Using these filtering criteria, we identified an Aeromonas hydrophila isolate which was positive for *bla*_CTX-M-15_ and one E. coli isolate that was positive for *bla*_CTX-M-15_ and *bla*_NDM-1_. Neither *bla*_CTX-M-15_ nor *bla*_NDM-1_ genes were detected in any of the other isolates. From this point on in the study, we referred to this E. coli isolate as E. coli PI7. To the best of our knowledge, this constitutes the first report of *bla*_NDM-1_ in a nonnosocomial environment in Saudi Arabia. In order to further evaluate the resistome and pathogenicity potential of this strain, a sequencing effort was carried out.

### Overall genomic size and traits of E. coli PI7.

**FIG 2:**
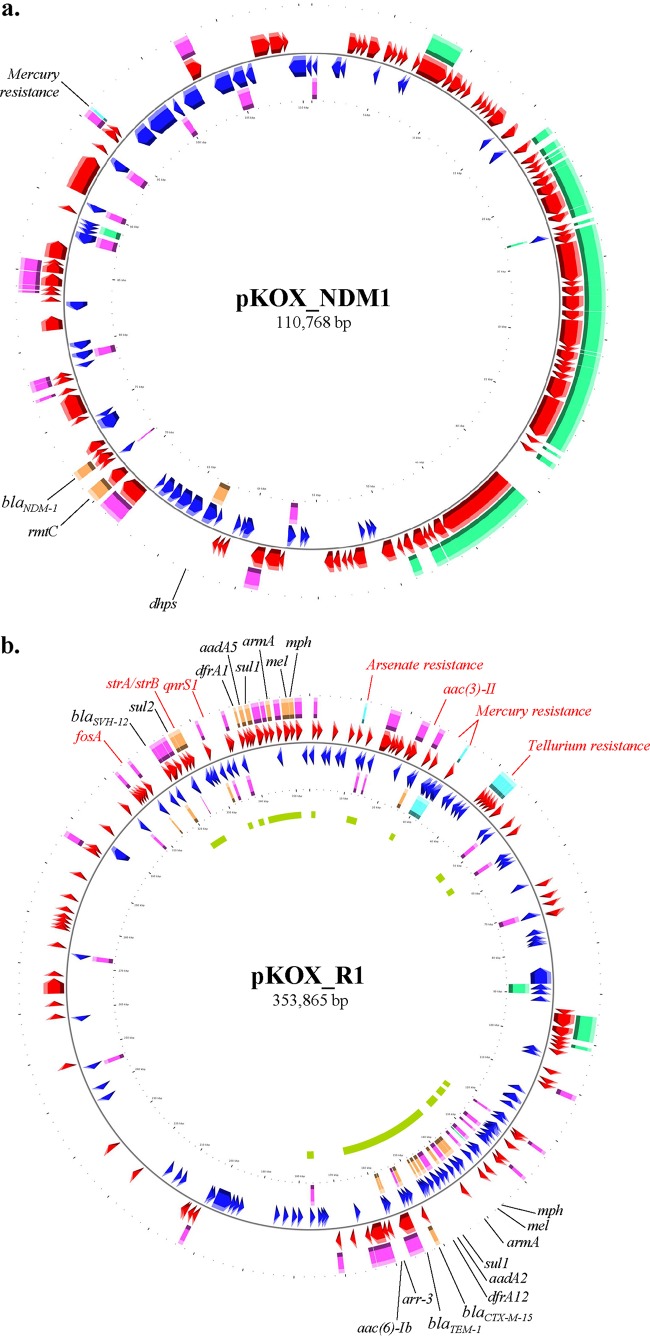
Genetic map of the plasmid carrying *bla*_NDM-1_ in E. coli PI7 (pKOX_NDM1) and contigs mapping against pKOX_R1 of Klebsiella oxytoca. Relevant characteristics related to antimicrobial resistance (orange), metal resistance (light blue), mobilization elements (purple), and plasmid maintenance and stability (green) are highlighted in the map. (a) pKOX_NDM1 was fully sequenced in this study and shares 99% sequence identity with the reported sequence for K. oxytoca (18). (b) pKOX_R1 was drawn based on the reported sequence for K. oxytoca (18). The inner green circle in the pKOX_R1 map represents the coverage of the contigs in this study mapping against pKOX_R1. Genes highlighted in red type represent those encoding relevant traits mapping outside the contigs found in this study.

The E. coli PI7 genome is 4.6 Mb in size and has an average GC content of 50.85%. It harbors 108 noncoding RNAs (ncRNAs) and 4,320 open-reading frames (ORFs) corresponding to 842 and 3,478 hypothetical and nonhypothetical proteins, respectively. MLST analysis based on seven housekeeping genes on the chromosomal genome revealed that E. coli PI7 belongs to ST101. Besides the chromosomal genome, E. coli PI7 also carries one plasmid of 110 kb from the IncF incompatibility group and a secondary IncA/C plasmid that was partially identified (Fig. 2). The IncF plasmid shared high similarity (>97%) with Klebsiella oxytoca pKOX_NDM, while the other plasmid in E. coli PI7 contains contigs that showed high BLAST hit scores with pKOX_R1 of Klebsiella oxytoca (18).

### Resistance repertoire encoded on the chromosome and plasmid.

Both the chromosome and the plasmid of E. coli PI7 carry a wide spectrum of ARGs. The predominant traits encoded on the plasmidic material of E. coli PI7 were carried by 17 genes associated with antibiotic resistance to aminoglycosides, beta-lactams, tetracyclines, fluoroquinolones, sulfonamides, macrolides, and trimethoprim (Fig. 2). More importantly, the IncF plasmid that shared high similarity with pKOX_NDM1 carries a copy of the New Delhi metallo-beta-lactamase (NDM) gene (see Table S2 in the supplemental material), which confers resistance to a broad spectrum of beta-lactams, including carbapenem antibiotics. On the other hand, most of the ARGs occurred in contigs that mapped against pKOX_R1, including a copy of *bla*_CTX-M-15_, which is an extended-spectrum beta-lactamase gene of clinical relevance (31).

Besides *bla*_NDM-1_, three other beta-lactamase genes, prevalent in other pathogenic and commensal E. coli isolates, were also identified in the chromosomal genome of E. coli PI7 (see Table S3 in the supplemental material). Multidrug efflux pumps were also identified as the main mechanism for antibiotic resistance in the chromosome, with 44 of the 47 antibiotic resistance loci belonging to this category (see Table S4 in the supplemental material). The list of multidrug efflux pumps was comprised of ABC transporters, such as the macrolide-specific efflux complex MacAB, the resistance-nodulation-division RND-type multidrug resistance locus, including the *acrAB* and *mdtABCD* operons, major facilitator superfamily transporters such as MdfA, and several other tripartite multidrug resistance systems.

The antimicrobial resistance genetic traits encoded on the chromosomal and plasmidic material of E. coli PI7 were further verified by MIC tests. E. coli PI7 was observed to be highly resistant to 256 μg/ml of gentamicin, ampicillin, kanamycin, ceftazidime, and sulfamethoxazole-trimethoprim, 128 μg/ml of chloramphenicol and erythromycin, and 64 μg/ml of tetracycline and meropenem but susceptible to 50 μg/ml of sodium azide.

### Virulence-associated traits.

**FIG 3:**
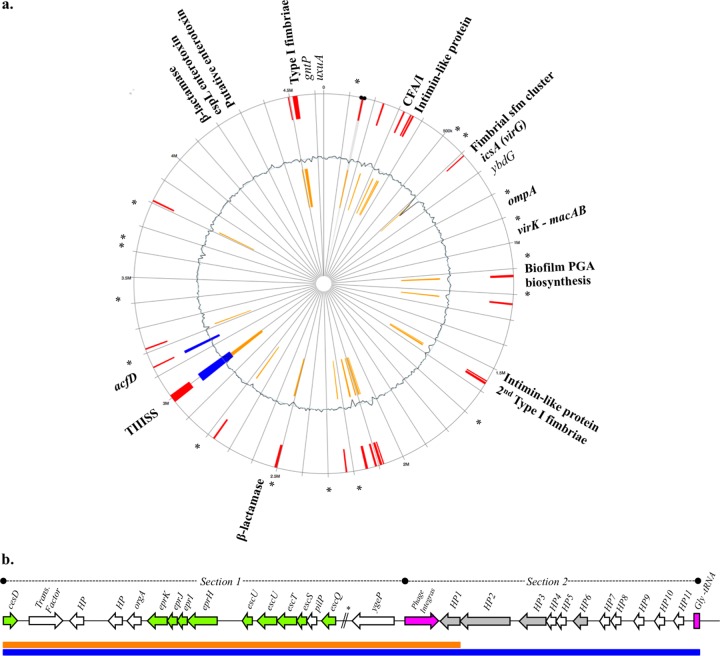
(a) Mapping of genomic islands, pathogenicity factors, and antibiotic resistance loci in the E. coli PI7 genome. Yellow and blue lines represent genomic islands predicted by SIGI-HMM and IslandPath/DIMOB, respectively. Red lines represent the genomic locations of all predicted islands. Asterisks correspond to locations of antibiotic efflux pumps. (b) T3SS gene cluster in E. coli PI7. Green arrows represent structural T3SS genes, pink arrows represent genetic elements associated with gene mobility, and gray arrows represent genes encoding hypothetical proteins (HPs) predicted by Secretome 1.1 for noncanonical secretion signals.

Twenty-four genomic islands (GIs) ranging from 4 to 32 kb were detected in the E. coli PI7 genome. These islands harbor several metabolic and pathogen-associated characteristics, with the most relevant traits depicted in Fig. 3a. The repertoire of pathogenic traits in E. coli PI7 includes the following: (i) the colonization fimbrial antigen I (CFA/I), which has been linked with enterotoxigenic E. coli (ETEC) pathotypes (32); (ii) surface protein OmpA, typically required for cell adhesion and invasion (33); (iii) two intimin-like proteins associated with enterohemorrhagic and enteropathogenic E. coli (EHEC and EPEC, respectively) pathotypes (34); (iv) surface protein IcsA (VirG) required for actin-dependent movement and inter- or intracellular spread in enteroinvasive E. coli (EIEC) and Shigella flexneri (35, 36); (v) an accessory colonization factor (AcfD) previously identified in Vibrio cholerae which facilitates efficient intestinal colonization (37); and (vi) a secondary copy of the type 1 fimbriae located ectopically in a GI and showing >99% amino acid sequence identity with fimbrial clusters of pathogenic E. coli O157:H7 strain EDL933.

### T3SS in the pathogenic genomic island.

The largest genomic island (GI) was 32 kb in size and was independently predicted by SIGI-HMM and IslandPath/DIMOB. This GI encodes a type III secretion system (T3SS) and a set of hypothetical proteins downstream of the structural T3SS (Fig. 3b). Section 1 of the T3SS gene cluster (Fig. 3b) is highly similar to the locus of enterocyte effacement (LEE) in E. coli O157:H7, except that a 9-kb region located upstream of *ygeP* is missing in the putative secretion system of E. coli PI7. Nevertheless, this same deletion is also present in other pathogenic but less virulent E. coli strains, such as EHEC strain O26:H11 and ETEC strains B7A and E24377A (see Fig. S2 in the supplemental material). The T3SS identified in the E. coli PI7 genome shared up to 99% nucleotide identity with that present in the previously mentioned strains. Similar to E. coli O26:H11 and B7A, E. coli PI7 also presents a set of hypothetical proteins (HPs) encoded by genes from *ygeP* to the glycine-tRNA (Fig. 3b). No known functions are assigned to these HPs. However, 3 of the 11 HPs were predicted to have noncanonical secretion signals when analyzed by Secretome 1.1 (*P* > 0.8).

### *In vitro* cell invasiveness.

**FIG 4:**
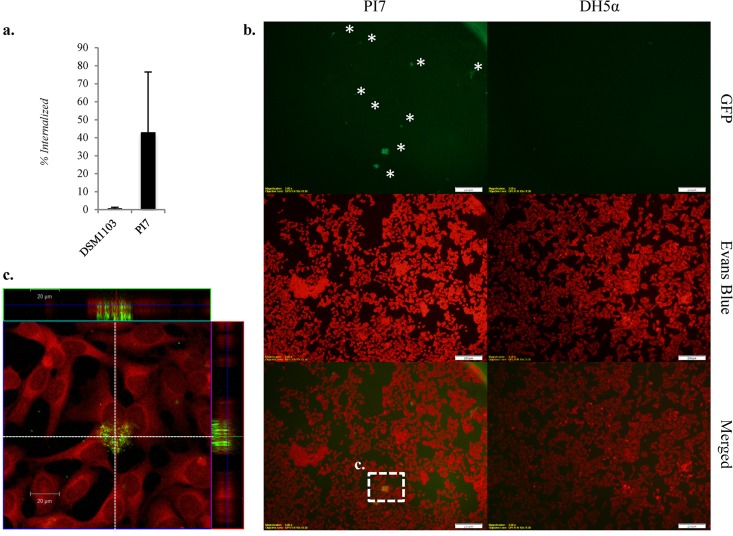
*In vitro* internalization of E. coli PI7, DSM 1103, and DH5α in HeLa cell cultures. (a) Cell internalization of E. coli PI7 versus that of E. coli DSM 1103; (b) confocal microscopy of HeLa cell cultures infected with GFP-tagged E. coli PI7 and GFP-tagged E. coli DH5α (magnification, ×20); (c) z-stack of highlighted box in panel b corresponding to HeLa cells infected with E. coli PI7 (magnification, ×60). Asterisks highlight green fluorescent clusters.

As the genome sequencing revealed several pathogenicity traits associated with cell adhesion and invasion, E. coli PI7 was tested *in vitro* for its ability to internalize HeLa cells. A commensal strain of E. coli DSM 1103 was used for comparison, as genomic sequencing of this strain does not show traits for internalization (38). On average, 43% of the inoculated E. coli PI7 cells showed internalization into HeLa cells after 4 h of exposure (Fig. 4a). In addition, the confocal microscopy results confirmed that E. coli PI7 shows enhanced adhesion/internalization in HeLa cell cultures compared to the nonpathogenic control E. coli DH5α, which did not exhibit adhesion or internalization (Fig. 4b and c).

## DISCUSSION

The combination of cultivation and molecular biology-based approaches to identify emerging microbial contaminants, especially in developing countries like Saudi Arabia, has provided an interesting outlook on the occurrence of ARB that may be present in untreated wastewater. In this study, *bla*_NDM-1_ genes were ubiquitously detected in untreated wastewater entering a Jeddah WWTP throughout the monitored period (Fig. 1) and at an abundance ranging from 1.6 × 10^4^ to 2 × 10^5^ copies/m^3^. The ubiquitous presence of *bla*_NDM-1_ genes in the wastewater may arise from the global travelers entering Jeddah each year. Jeddah serves as the main point of entry for up to 2 million international Hajj pilgrims per year. Many of these pilgrims come from developing countries where the use of antibiotics is not properly monitored, which can facilitate the selection of multidrug-resistant bacteria. To illustrate, NDM was first discovered in a Swedish patient of Indian origin who had traveled and stayed in India (6). A similar incident was also reported in the United Kingdom, where a patient of Indian origin was infected with NDM-positive E. coli after his visit to India (39). In the present study, although international pilgrims may have contributed to the presence of *bla*_NDM-1_ in the wastewater, there was no apparent spike in the abundances of this gene during Hajj timings (October 2012 and October 2013). These results suggest that while Hajj would see a massive influx of pilgrims during a short period of time, the pilgrims that come throughout the year for Umrah, a shorter version of pilgrimage, may be contributing more toward the constant presence of the *bla*_NDM-1_ genes in the wastewater.

Alternatively, the results may suggest an existing ubiquity of *bla*_NDM-1_ genes that are already present in the local wastewater. The high environmental circulation of these genes in Jeddah wastewater may not be an isolated event, as an earlier study also found a ubiquitous presence of *bla*_NDM-1_ genes throughout the wastewater treatment process in China (10). However, most of the existing studies that track the presence of *bla*_NDM-1_ genes in wastewater relied on molecular biology-based approaches to detect the genes and did not further determine the identity of the associated bacterial host and their genomic contents. To further track the associated bacteria that carry *bla*_NDM-1_ genes, a cultivation effort was carried out in this study to recover *bla*_NDM-1_-positive bacteria on MacConkey agar plates. Through this effort, 24 bacterial isolates that were resistant to meropenem were recovered, but most of them, except for 2 isolates, did not test positive for genes targeted by any of the tested ARG primers. Potentially new resistance mechanisms may be present in most of these isolates, which warrants a future study to characterize them in detail. For the remaining two isolates, one was identified to be *bla*_CTX-M-15_-positive Aeromonas hydrophila, while the other was identified to be Escherichia coli positive for both *bla*_NDM-1_ and *bla*_CTX-M-15_. Characterization of the E. coli isolate was emphasized, as its approximate isolation frequency of 3 × 10^4^ CFU/m^3^ of wastewater coincides with the detected copy numbers of *bla*_NDM-1_ genes in the wastewater (Fig. 1).

Due to Saudi Arabia's general lack of sanitation infrastructure to provide efficient waste management and containment, the presence of this bacterium in untreated wastewater could lead to the potential dissemination of this ARB and its associated ARGs into the environment. Although there is a strong correlation between resistome and phylogeny in soil and activated sludge microbial communities, which would theoretically infer low horizontal ARG transfer in the environment (40, 41), this study pointed out the presence of near-complete plasmid sequences harboring the ARGs in E. coli PI7. Most importantly, the identified plasmids are densely populated with insertion sequences and other mobile genetic elements. This ARG architecture has been shown to facilitate the dissemination of ARGs across environments (41, 42). Therefore, there is a high possibility that the microbial communities in the soil or activated sludge might become reservoirs of such genes after the decay or lysis of E. coli PI7 has taken place after dissemination in the environment.

To understand the extent of risks arising from this bacterium, its genome was characterized. The genome annotation revealed an extensive plethora of genes with resistance against a wide spectrum of antibiotics, encoded on two high-molecular-weight plasmids. In addition to the plasmidic ARGs, efflux pumps were the prevalent antimicrobial resistance mechanism in the chromosome. Besides their role in antimicrobial tolerance, efflux pumps have also shown to confer resistance to natural substances produced by the host, such as bile, hormones, and defense molecules, and some members of the RND family have even been shown to facilitate colonization and persistence on the host (43). In addition to these traits, E. coli PI7 also encodes multiple heavy metal efflux pumps and detoxification enzymes, as well as UV protection proteins that might favor its survival and persistence in the secondary habitat outside the host.

E. coli PI7 also showed virulence-associated traits, including colonization fimbriae, surface proteins, and intimin-like proteins, that would likely explain the successful internalization of this bacterium into HeLa cells, compared to that of E. coli DSM 1103 and DH5α. Genome characterization of E. coli PI7 further showed that this strain encodes a T3SS, which is a critical pathogenicity trait, as it allows the translocation of effector proteins that modulate the response of the host cell (44). Enterotoxins are a group of effectors secreted by the T3SS that are necessary for the development of EHEC and ETEC phenotypes (34). E. coli PI7 has two enterotoxin genes located outside the T3SS GI—one putative and one identified as *espL* (Fig. 3a). No other effector genes were detected during the annotation process or by endpoint PCR (i.e., *nleB*, *nleC*, *nleH1*, and *nleE*) (see Fig. S3a and b in the supplemental material).

Based on the repertoire of virulence-associated features identified in the genome of E. coli PI7, it is highly likely that this bacterium is an opportunistic pathogenic strain. However, it was difficult to classify it into any of the commonly described pathotypes (i.e., EHEC, EPEC, ETEC, or UPEC), as it carries a mosaic of traits present in several of these clusters. This observation is in agreement with an earlier study that noted the large genome plasticity observed in E. coli, which in turn complicated the ability to categorize the various clusters of pathogenic E. coli strains into sharply delineated groups (34). Nevertheless, given that E. coli PI7 is identified as ST101 phylogroup B1 by MLST, a serotype commonly associated with nosocomial infections (45 – 49), it is likely that E. coli PI7 ended up in the wastewater after dissemination from a host carrier or infected patient. Although the exact origin cannot be traced, it is of potential public health concern that a bacterium with such virulence-associated traits would remain viable and could be resuscitated upon cultivation. In addition to the clinical significance of this particular sequence type, isolates of phylogroup B1 have been associated with prolonged environmental persistence, suggesting that these genotypes might have an adaptive advantage in the habitat outside the host (50).

To date, there had been no known reports of *bla*_NDM-1_-positive E. coli isolated from nosocomial samples in Saudi Arabia. However, *bla*_NDM-1_-positive K. pneumoniae had been identified (17, 51). In one study which aimed to establish the role of local transmission versus possible pathogen import due to global travel, it was determined that patients carrying *bla*_NDM-1_-positive K. pneumoniae had no prior documented foreign exposure, hence suggesting a high rate of autochthonous infections (52). Even though the isolation frequency for E. coli PI7 (i.e., 10^4^ CFU/m^3^) matches the gene abundance data measured by qPCR (1.6 × 10^4^ copies/m^3^ for October 2013), it is likely that the *bla*_NDM-1_ gene may also be carried by other bacteria like K. pneumoniae. Regardless, this is, to the best of our knowledge, the first report of a *bla*_NDM-1_-positive E. coli isolated from a nonnosocomial environment in Saudi Arabia and may set a priority concern for the need to establish improved surveillance for carbapenem-resistant E. coli in this country and nearby regions.

The environmental incidence of this clinically important ARG in Saudi Arabia and other regions in the world means that it is imperative to conduct future studies on the environmental fate and persistence of this gene. We believe that E. coli PI7 might serve as an appropriate model for these types of studies, particularly considering that this bacterium of apparent clinical origin was recovered from the environment. The genome sequencing of this strain revealed an ARG architecture that facilitates horizontal gene transfer, as well as pathogenicity and environmental persistence traits that would favor the survival of this bacterium in the host and the outside environment.

## Supplementary Material

Supplemental material
