# Supplementary material for: Isolation and Characterization of NDM-Positive Escherichia coli from Municipal Wastewater in Jeddah, Saudi Arabia
Source: Antimicrob Agents Chemother. 2016 Aug 22;60(9):5223–31. doi: 10.1128/AAC.00236-16 (PMC4997845; doi:10.1128/AAC.00236-16)
Supplement: Supplemental material [file supp_60_9_5223__index.html]

Isolation and Characterization of NDM-Positive Escherichia coli from Municipal Wastewater in Jeddah, Saudi Arabia — Supplemental material 

# Isolation and Characterization of NDM-Positive Escherichia coli from Municipal Wastewater in Jeddah, Saudi Arabia

## Supplemental material

- Supplemental file 1 -

  Supplemental Text S1 to S7, Figures S1 to S3, and Tables S1 to S4.

  PDF, 1.7M
