# Supplementary material for: Isolation and Characterization of NDM-Positive Escherichia coli from Municipal Wastewater in Jeddah, Saudi Arabia
Source: Antimicrob Agents Chemother. 2016 Aug 22;60(9):5223–31. doi: 10.1128/AAC.00236-16 (PMC4997845; doi:10.1128/AAC.00236-16)
Supplement: Supplemental material [file AAC.00236-16_zac009165451so1.pdf]

Supplementary information for

Isolation and characterization of NDM-positive *Escherichia coli* from municipal wastewater in Jeddah, Saudi Arabia

1. qPCR optimization and validation. bla_{NDM-1} copy numbers were determined by absolute quantification qPCR, using the NDM-154-Forward: 5'- ATTAGCCGCTGC ATTGAT-3' and NDM-154-Reverse: 5'- GGCATGTCGAGATAGGAAGT-3' primer set (154 bp amplicon) (1), and the NDM-22: 5'-/6-FAM/AGACATTCG/ZEN/GTGCGA GCTGGCGGA/IABkQ TaqMan probe (this study). qPCR was performed on a 7900HT Fast Real-Time PCR system (Applied Biosystems), using the TaqMan® Fast Advanced Master Mix (Life Technologies). Primer and probe concentrations were optimized as described by the manufacturer, in order to obtain the best signal to noise ratio. Optimized concentrations were determined to be 300 nM for each primer and 250 nM for the probe. Supplementary Figure 1 shows the amplification plot at the optimized primer/probe concentrations, and the standard curve generated. At these primer/probe concentrations the NTCs did not show fluorescent signal above detection threshold. The standard curve was generated by cloning a 640 bp bla_{NDM-1} fragment into the pCR® 2.1-TOPO cloning vector (Thermo Fisher Scientific). The 640 bp fragment was amplified using the 5'-TAG TGC TCA GTG TCG-3' forward and 5'- CAT TAG CCG CTG CA-3' reverse primer pair. Each standard curve contained 5 reference points, corresponding to 10^8, 10^6, 10^4, 10^3 and 10^2 bla_{NDM-1} copy numbers per µL.

2. Meropenem-EDTA combined disk diffusion test. To further screen for the presence of bla_{NDM-1} gene, *E. coli* PI7 was grown overnight (ON) on LB broth supplemented with meropenem (8 µg/mL) at 37°C and 200 rpm. Subsequently, the ON cultures where diluted to an OD_{600} of 0.07 and the diluted culture was spread-plated onto a Mueller-Hinton agar. Once the bacteria were plated, 4 sterile diffusion discs were placed on each quadrant of the plate. Each of the sterile diffusion disc was spotted with 10 µL of either (i) 0.4 µL of 25 mg/mL meropenem, (ii) 3 µL of 0.5 µM EDTA, (iii) 0.4 µL of 25 mg/mL meropenem + 3 µL of 0.5 µM EDTA, or (iv) sterile water. Zinc ions serve as cofactors

for NDM activity (2); chelation by EDTA would result in the inhibition of the hydrolytic activity. Hence, in bacterial isolates positive for bla_{NDM-1}, a zone of inhibition would be anticipated near the sterile disc spotted with EDTA-meropenem and no zones of inhibition are anticipated near the remaining three discs. Isolates that exhibit such phenotypic traits were further confirmed for bla_{NDM-1} by end-point PCR using the primer pairs 5'-CATTAGCCGCTGCATTGA-3' forward and 5'-TAGTGCTCAGTGTCG-3' reverse primer pair (1). Gene sequences were obtained by Sanger Sequencing and subsequently matched against the National Center for Biotechnology Information (NCBI) nucleotide sequences database using BLASTN to determine the presence of bla_{NDM-1}.

3. Processing of genome and plasmid sequences. Raw sequencing reads were subjected to data trimming and filtering. In the first step, we check for the presence of adapter sequence and removed the adapter sequence from the reads. Bases at the 3' end that fell below a quality of 20 were trimmed off. Reads with average quality score of 20 were discarded. Finally, reads with at least 50 bases length were retained for further analysis. Two de-novo assemblers, namely CLC genomics workbench and SOAPdenovo were used. k-mer sizes of 40 and 50 were used for CLC-Genomic workbench and k-mer sizes 31, 41, 51, 61, 71, 81 and 91 were used for SOAPdenovo. Then, the output of all the assemblies were combined into a large super-set of sequences. Contig that contained "N" was split into contigs by removing the N's. In order to reduce the redundancy, merged contigs were first processed by CD-HIT-EST with 100% identity to remove identical fragments. The contigs were then sorted based on their size. Sorted contigs were used to create preliminary scaffolds. Each contig was used to search against the remaining contigs to find an overlap at the ends of the contigs. If the contig finds at least 50 bases overlap with another contig at the ends, then both contigs were merged to form a single contig. Then, the merged contig was used to search against the remaining unmerged contigs to find overlapped contigs. This iterative process of overlap determination and contig assembly is repeated until there are no remaining overlaps among the contigs. Subsequently, we ordered and oriented these contigs into larger units (scaffolds), which usually requires a reference genome. Since we do not have complete reference genome for our organism, we used another closely-related bacterial genome as a reference. To

identify a closely-related reference microorganism, we performed BLAST search for each contig against NCBI bacterial genome database. Then, we calculated alignment coverage for each bacterial genome from total number of bases aligned in the genome divided by the size of the genome. Finally, we selected NC_011741 (*Escherichia coli* IAI1 chromosome- complete genome) as a reference genome. There are two reasons for selecting NC_011741 genome. First, the size of NC_011741 genome is similar to the genome we have sequenced. Second, more than 95% of the genome is aligned with our contigs.

The contigs were sorted based on their alignment position on the IAI1 chromosome genome. Finally the gap-size between two contigs was calculated and evaluated. If the calculated value is negative and an overlap was found, contigs are merged. On the other hand, positive value indicates that there is a gap between the contigs. These gaps are closed by mapping the raw reads that span gaps between two scaffolds. If the gap is not connected by any read, then a gap between contigs using one or more undefined ‘N’ nucleotides depending on the gap-size were inserted.

The same plasmid assembly process was performed with the exception that the reference plasmids selected was pKDO1 (GenBank no. JX424423) from *Klebsiella pneumoniae*. The reason for this was that more than 95% of the plasmid is aligned with our plasmid contigs.

4. Minimum inhibitory concentrations determination. Minimum inhibitory concentrations (MICs) of *E. coli* PI7 were determined by microtiter broth dilution method under aerobic condition. A wide range of antibiotics that include gentamicin, ampicillin, kanamycin, ceftazidime, erythromycin, sulfamethoxazole-trimethoprim, chloramphenicol, tetracycline and meropenem was tested. 195 µL of LB broth with antibiotic concentrations of 0 (i.e., positive control), 8, 32, 64, 128 and 256 µg/mL were individually inoculated with 5 µL of overnight *E. coli* PI7 culture, and pipetted into separate wells of a 96-well microtiter plate. Negative controls comprising of only LB with no antibiotics and bacterial culture were also prepared into separate wells of the same microtiter plate. The prepared microtiter plates were incubated for 8 h at 37 °C under aerobic conditions. Endpoint optical density at 600 nm (OD_{600}) was measured

using the Spectromax 340pc microplate spectrophotometer (Molecular Devices, Sunnyvale, CA, USA). MIC of a particular antibiotic was determined based on the concentration required to achieve ≥ 70% growth inhibition as compared to the positive control. No change in the OD_{600} measurement was observed in the negative controls.

5. GFP-tagged strain construction. The ZsGreen gene (GeneArt Gene Synthesis) was flanked with *EcoR*I and *BamH*I sites by PCR using the following primers GFP-F, 5'-CCGGAATTCGATGGCTCAGTCAAAG-3'; GFP-R, 5'-CGCGGATCCTCAGGGCAATGCAG-3' (the underlined sequences correspond to *EcoR*I and *BamH*I sites). The PCR amplification was done with Q5 hot start high-fidelity DNA polymerase (New England Biolabs, Ipswich, MA, USA) under the following conditions: initial denaturation step at 98 °C x 30 s, followed by 30 cycles of 98 °C x 10 s, 70 °C x 30 s , 72 °C x 20 s and final extension at 72 °C x 2 min. The PCR products were digested with *EcoR*I and *BamH*I, and subcloned into PSTV28 vector (Takara) belonging to Inc B group. The recombinant plasmid PSTV28/GFP was confirmed by sequencing and separately transformed into *E.coli* Mach1™ T1 (Thermo Fisher Scientific, Carlsbad, CA, USA) and *E.coli* PI-7. All PCR products were amplified under the following conditions: denaturation at 98 °C x 30 s followed by 30 cycles 98 °C x 10 s, 65 °C x 1 min and 72 °C x 1 min. Expression of GFP and plasmid stability was confirmed by fluorescence microscopy and flow cytometry after approximately 4000 generations (data not shown).

6. Invasiveness assay. HeLa cells were obtained from ATCC (CCL-2) and were maintained in Invitrogen Dulbecco's Modified Eagle's medium (DMEM) with 10 % fetal bovine serum (FBS) (Thermo Fisher Scientific, Carlsbad, CA, USA) supplemented with 1% penicillin/streptomycin (Thermo Fisher Scientific, Carlsbad, CA, USA). Cells were serially passaged in 75 cm^2 vent-cap sterile non-pyrogenic polystyrene tissue culture flasks (VWR, Radnor, PA, US) and incubated at 37 ^oC with 5 % CO_2. HeLa cells were then seeded at a density of 3 x 10^5 cells/well into 8 wells of a Costar® 24-well clear tissue culture-treated plate. Each well contained DMEM with 10 % FBS supplemented with 1% penicillin/streptomycin. The cell cultures were then incubated overnight in

37 ^{o}C, 5% CO_2. Single colonies of *E. coli* PI7, *E. coli* DSM1103, GFP-tagged *E. coli* PI7 and GFP-tagged *E. coli* DH5α were picked and inoculated in 5 mL of LB broth for incubation at 37 ^{o}C for 10 h or till the culture achieved an OD_{600} of 0.8. *E. coli* DSM 1103 and *E. coli* DH5α were used as non-pathogenic controls.

Bacterial cells were pelleted at 5000 g for 10 minutes and resuspended in 1.2 mL of DMEM. The media for the HeLa cells on the 24-well plate was replaced with 300 µL of the either *E. coli* suspension (n = 4 for each strain) or mocked inoculated with sterile DMEM (n = 4 for plating assays and n = 4 for confocal microscopy assays). HeLa cells were then further incubated in the presence of the bacteria cells for 2 h in 37 ^{o}C, 5 % CO_2. After incubation, wells inoculated with GFP-tagged *E. coli* were processed for confocal microscopy and wells inoculated with non GFP-tagged strains treated with mutanolysin as described in the main text.

7. Fixation and staining for confocal microscopy. After the completion of the three washing steps with 1X PBS, mammalian cells were fixed with acetone for 10 minutes at 4 °C. Cells were then labeled with Evans blue (Milipore 5008) for 30 minutes at 37 °C. Cells were visualized using Zeiss LSM 710 upright confocal microscope using appropriate machine settings. Z-stack images were taken with the Zen Black software at 40x magnification.

References

1. **Cunningham SA, Noorie T, Meunier D, Woodford N, Patel R.** 2013. Rapid and simultaneous detection of genes encoding Klebsiella pneumoniae carbapenemase (blaKPC) and New Delhi metallo-β-lactamase (blaNDM) in Gram-negative bacilli. Journal of Clinical Microbiology **51:**1269-1271.
2. **Nordmann P, Poirel L, Walsh TR, Livermore DM.** 2011. The emerging NDM carbapenemases. Trends Microbiol **19:**588-595.

a.

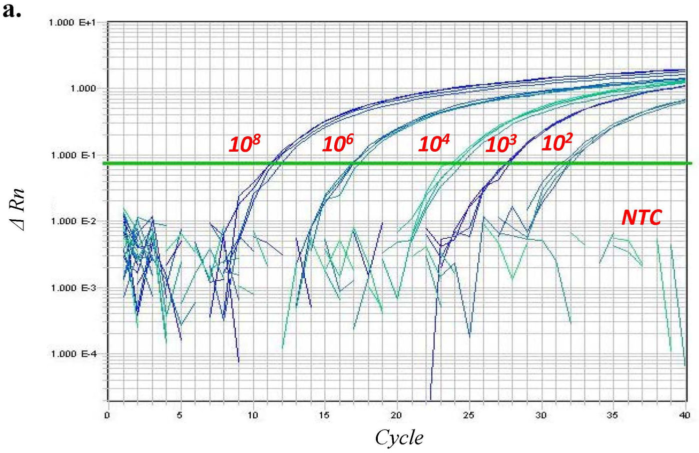

b.

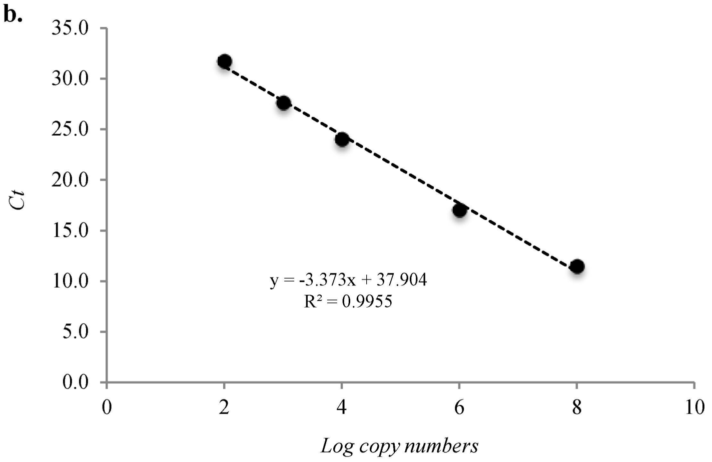

Supplementary Figure 1. a. amplification plot and **b.** standard curve generated with the optimized primer/probe concentrations. Amplification efficiency with this primer pair and TaqMan probe was 98%.

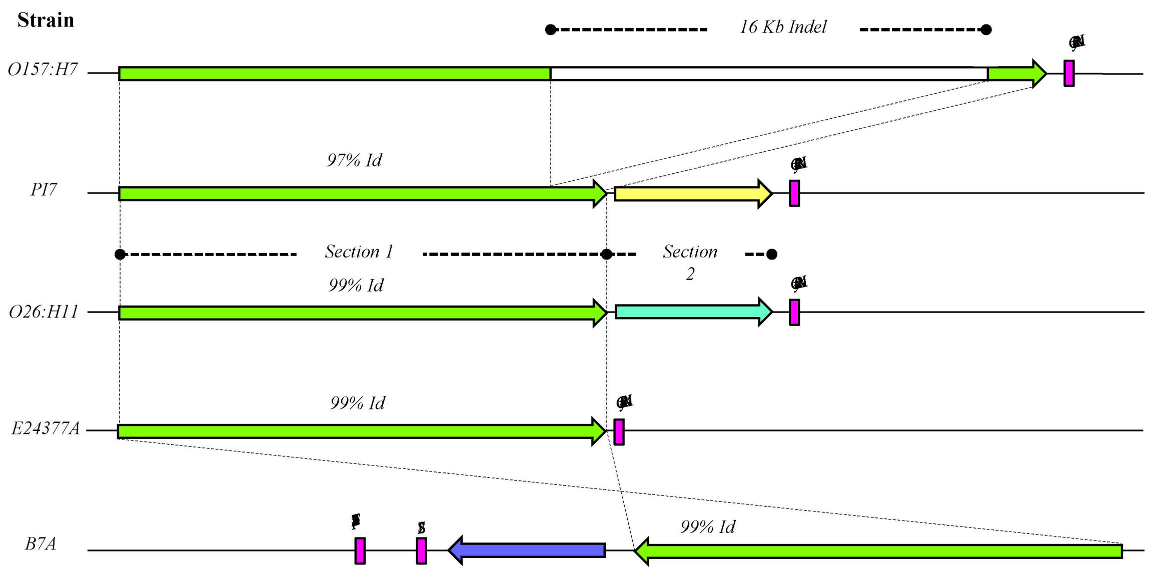

Supplementary Figure 2. Genomic rearrangements of the TIIISS in 5 *E. coli* strains. *E. coli* O157:H7 strain Sakai represents the complete LEE pathogenicity island. Section 2 was not conserved between any of the strains analyzed here.

a.

Name	*Sequence*	*Amplicon size (bp)*
nleB_Fwd	*CACAAATGCGGCAAGGATAGAG*	*515*
nleB_Rev	*CATTACGACGATCCACATGC*	
nleC_Fwd	*AGCGAATACCACTTTCCGCA*	*536*
nleC_Rev	*TCCCAGTTCTTGAGCGACAC*	
nleH1_Fwd	*GCCAGAGTTACCGAGTGTGG*	*448*
nleH1_Rev	*ACTCCATGAACGGTCGGAAA*	
nleE_Fwd	*TAATACTCAGGGCGTGTCCC*	*462*
nleE_Rev	*CGTTGTTCTCCTAGAGGGCTG*	

b.

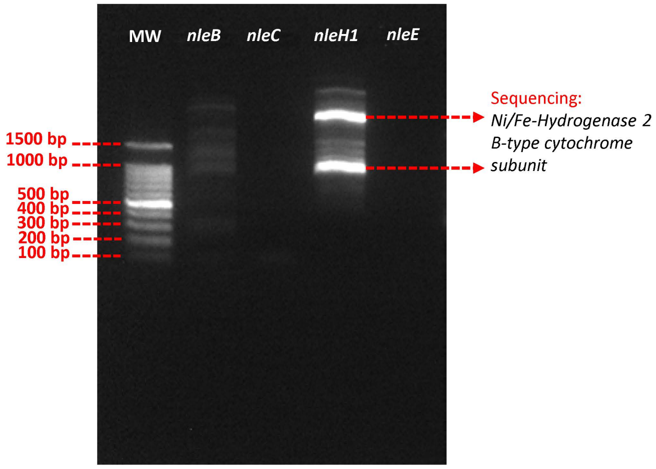

Supplementary Figure 3. a. Primer sets used for the amplification of effector gene *nleB*, *C*, *H1* and *E*. Thermal cycling conditions were: 95°C x 5 min; 40 cycles of 60°C s x 60s, 72°C x 1:30 min; and final elongation 72°C x 5 min. **b.** PCR gel image for effector genes. Sanger sequencing showed that upper and lower correspond to interference by *Ni/Fe-Hydrogenase 2 B-type cytochrome.*

Supplementary Table 1. Phylogenetic identities of isolates recovered from 30 mL of untreated wastewater.

**Isolate name**	**Best-matched identity based on 16S rRNA gene**	**Identity similarity (%)**	**Max score**
PI1	*Aeromonas caviae*	100	1801
PI2	*Aeromonas hydrophila*	99	1793
PI3	*Aeromonas caviae*	100	1920
PI4	*Aeromonas caviae*	99	1837
PI5	*Aeromonas caviae*	99	1790
PI6	*Aeromonas caviae*	99	1795
PI7	*Escherichia coli*	99	1833
PI8	*Aeromonas veronii*	99	1777
PI9	*Aeromonas veronii*	100	1825
PI10	*Aeromonas hydrophila*	100	1513
PI11	*Pseudomonas putida*	100	1775
PI12	*Pandoraea apista*	100	1772
PI13	*Bacillus* sp.	100	610
PI14	*Bacillus* sp.	100	344
PI15	*Enterococcus hirae*	100	1037
PI16	*Bacillus* sp.	100	776
PI17	*Bacillus* sp.	100	800
PI18	*Bacillus clausii*	100	250
PI19	*Bacillus* sp.	100	564
PI20	*Bacillus* sp.	100	507
PI21	*Pandoraea* sp.	100	1862
PI22	*Pseudomonas sp.*	100	1903
PI23	*Pandoraea* sp.	100	1436
PI24	*Pandoraea* sp.	99	1783

Supplementary Table 2. NDM-1 location and DNA sequence.

Plasmid	Size (bp)	Strand	Sequence (5’-3’)
IncF plasmid	915	Plus	TTGAATTCGCCCCATATTTTTGCTACAGTGAACCA<br>AATTAAGATCATCTATTTACTAGGCCTCGCATTTG<br>CGGGGTTTTTAATGCTGAATAAAAGGAAAACTTG<br>ATGGAATTGCCCAATATTATGCACCCGGTCGCGA<br>AGCTGAGCACCGCATTAGCCGCTGCATTGATGCT<br>GAGCGGGTGCATGCCCGGTGAAATCCGCCCGAC<br>GATTGGCCAGCAAATGGAAACTGGCGACCAACG<br>GTTTGGCGATCTGGTTTTCCGCCAGCTCGCACCG<br>AATGTCTGGCAGCACACTTCCTATCTCGACATGC<br>CGGGTTCGGGGCAGTCGCTTCCAACGGTTTGATC<br>GTCAGGGATGGCGGCCGCGTGCTGGTGGTCGATA<br>CCGCCTGGACCGATGACCAGACCGCCCAGATCCT<br>CAACTGGATCAAGCAGGAGATCAACCTGCCGGTC<br>GCGCTGGCGGTGGTGACTCACGCGCATCAGGACA<br>AGATGGGCGGTATGGACGCGCTGCATGCGGCGG<br>GGATTGCGACTTATGCCAATGCGTTGTCGAACCA<br>GCTTGCCCCGCAAAAGGGGATGGTTGCGGCGCA<br>ACACAGCCTGACTTTCGCCGCCAATGGCTGGGTC<br>GAACCAGCAACCGCGCCCAACTTTGGCCCGCTCA<br>AGGTATTTTACCCCGGCCCCGGCCACACCAGTGA<br>CAATATCACCGTTGGGATCGACGGCACCGACATC<br>GCTTTTGGTGGCTGCCTGATCAAGGACAGCAAGG<br>CCAAGTCGCTCGGCAATCTCGGTGATGCCGACAC<br>TGAGCACTACGCCGCGTCAGCGCGCGCGTTTGGT<br>GCGGCGTTCCCCAAGGCCAGCATGATCGTGATGA<br>GCCATTCCGCCCCCGATAGCCGCGCCGCAATCAC<br>TCATACGGCCCGCATGGCCGACAAGCTGCGCTGA

Supplementary Table 3. Genomic coordinates and sequences of beta-lactamases in *E. coli* PI7

Genomic coordinates		Size (bp)	Protein sequence	Commensal Strains	Pathogenic Strains
2566409	2567701	1293	MLYLSLLAVSCSVSAAKYPVLTESSPEKAGFNVERLNQMDRWISQ QVDAGYPGVNLLIIKDNQIVYRKAWGAAKKYDGSVLMEQPVKAT TGTLYDLASNTKMYATNFALQKLMSEGKLHPDDRIAKYIPGFADSP NDTIKGKNTLRISDLLHHSGGFPADPQYPNKAVAGALYSQDKGQTL EMIKRTPLEYQPGSKHIYSDVDYMLLGFIVESVTGQPLDRYVEESIY RPLGLTHTVFNPLLKGFKPQQIAATELNGNTRDGVIHFPNIRTSTLW GQVHDEKAFYSMGGVSGHAGLFSNTGDIAVLMQTMLNGGGYGD VQLFSAETVKMFTTSSKEDATFGLGWRVNGNATMTPTFGTLASPQ TYGHTGWTGTVTVIDPVNHMAIVMLSNKPHSPVADPQKNPNMFES GQLPIATYGWVVDQVYAALKQK	K-12	O157:H7 (EHEC); 94-3024 (STEC); RM9387 (STEC); CFSAN029787 (EIEC); O104:H4 (EAEC)
4328162	4328920	759	MSLTLTLTGTGGAQGVPAWGCECAACARARRSPQYRRQPCSGVV KFNDAITLIDAGRHDLTDRWSPGSFQQFLLTHYHMDHVQGLFPLR WGVGDVIPVYGPPDEQGCDDLFKHPGLLDFSHTVEPFVVFDLQGL QVTPLPLNHSKLTFGYLLETAHSRVAWLSDTAGLPEKTLKFLLNNH PQVMVIDCSHPPRADAPRNHYDLNTVLALNQVIRSPQVILTHISHQF DAWLMENALPSGFEVGFDGMEIGVA	K-12; HS; W	026:H11 (EHEC); 94-3024 (STEC); CFSAN029787 (EIEC); O104:H4 (EAEC); 789 (Septicemic); ACN001 (Avian Pathogen); B7A (ETEC)
4391116	4392249	1134	MFKTTLCALLITASCSTFAAPQQINDIVHRTITPLIEQQKIPGMAVAV IYQGKPYYFTWGYADIAKKQPVTQQTLFELGSVSKTFTGVLGGEAI ARGEIKLSDPTTKYWPELTAKQWNGITLLHLATYTAGGLPLQVPDE VKSSSDLLRFYQNWQPAWAPGTQRLYANSSIGLFGALAVKPSGLSF EQAMQTRVFQPLKLNHTWINVPPAEEKNYAWGYREGKAVHVSPG ALDAETYGVKSTIEDMACWVRSNMNPRDINDKTLQQGIQLAQSRY WQTGDMYQGLGWEMLDWPVNPDSIINGSGNKIALAAHPVKAITPP TPAVRASWVHKTGATGGFGSYVAFIPEKELGIVMLANKNYPNPAR VAAAWQILNALQ	HS; W; IAI1; SE11	ECC-1470 (Cow Pathogen); FHI (STEC); O111:H- (STEC); O103:H2 (EHEC); O26:H11 (EHEC)

Supplementary Table 4. Genomic coordinates of efflux pumps in *E. coli* PI7 chromosome.

Locus	Genomic coordinates		Size (bp)	Gene description
1	142818	143744	927	ABC-type multidrug transport system, ATPase component
2	143741	144511	771	ABC-type multidrug transport system, permease component
3	518449	520230	1782	Multidrug resistance-like ATP-binding protein mdlB
4	532214	529065	3150	RND efflux system, inner membrane transporter CmeB
5	533322	532237	1086	Membrane fusion protein of RND family multidrug efflux pump
6	533572	534219	648	Transcription repressor of multidrug efflux pump acrAB operon, TetR (AcrR) family
7	860667	859561	1107	ABC transport system, permease component YbhR
8	861811	860678	1134	ABC transport system, permease component YbhS
9	863540	861804	1737	ABC transporter multidrug efflux pump, fused ATP-binding domains
10	864528	863533	996	Predicted membrane fusion protein (MFP) component of efflux pump, membrane anchor protein YbhG
11	865202	864531	672	Transcriptional regulator YbiH, TetR family
12	922286	923518	1233	Multidrug translocase MdfA
13	957787	958902	1116	Macrolide-specific efflux protein MacA
14	958899	960845	1947	Macrolide export ATP-binding/permease protein MacB (EC 3.6.3.-)
15	1153082	1151856	1227	Multidrug-efflux transporter, major facilitator superfamily (MFS) (TC 2.A.1)
16	1597205	1596540	666	Multiple antibiotic resistance protein MarC
17	1597417	1597851	435	Multiple antibiotic resistance protein MarR
18	1597871	1598254	384	Multiple antibiotic resistance protein MarA
19	1713558	1714931	1374	Multi antimicrobial extrusion protein (Na(+)/drug antiporter), MATE family of MDR efflux pumps
20	2193688	2194935	1248	Multidrug transporter MdtA
21	2194935	2198057	3123	Multidrug transporter MdtB
22	2198058	2201135	3078	Multidrug transporter MdtC
23	2201136	2202551	1416	Multidrug transporter MdtD
24	2202548	2203951	1404	Sensory histidine kinase BaeS
25	2203948	2204670	723	Response regulator BaeR
26	2319044	2317854	1191	MFS family multidrug transport protein, bicyclomycin resistance protein
27	2502878	2501340	1539	Inner membrane component of tripartite multidrug resistance system
28	2504041	2502878	1164	Membrane fusion component of tripartite multidrug resistance system
29	2798632	2799162	531	Transcription repressor of tripartite multidrug resistance system
30	2799289	2800461	1173	Multidrug resistance protein A (ErmA)
31	2800478	2802016	1539	Multidrug resistance protein B
32	3205513	3206994	1482	Type I secretion outer membrane protein, TolC precursor
33	3438862	3438200	663	Transcription repressor of multidrug efflux pump acrAB operon, TetR (AcrR) family
34	3439261	3440418	1158	RND efflux system, membrane fusion protein CmeA
35	3440430	3443534	3105	RND efflux system, inner membrane transporter CmeB
36	3630888	3628153	2736	ABC-type multidrug transport system, permease component
37	3660556	3661620	1065	Membrane fusion protein of RND family multidrug efflux pump
38	3661645	3664758	3114	RND efflux system, inner membrane transporter CmeB
39	3760386	3759250	1137	Multidrug resistance protein A
40	3775913	3774777	1137	Multidrug resistance protein A
41	3862275	3863459	1185	Multidrug resistance protein D
42	4321480	4320014	1467	Outer membrane component of tripartite multidrug resistance system
43	4323528	4321477	2052	Membrane fusion component of tripartite multidrug resistance system
44	4324559	4323528	1032	Inner membrane component of tripartite multidrug resistance system
